# Acetylcholinesterase inhibition ameliorates deficits in motivational drive

**DOI:** 10.1186/1744-9081-8-15

**Published:** 2012-03-20

**Authors:** Keri Martinowich, Kathleen M Cardinale, Robert J Schloesser, Michael Hsu, Nigel H Greig, Husseini K Manji

**Affiliations:** 1 Mood and Anxiety Disorders Program (MAP), National Institute of Mental Health (NIMH), National Institutes of Health (NIH), 35 Convent Drive, Building 35, Room 1C-1012, Bethesda, MD 20892-3711, USA; 2 Laboratory of Neuroscience, Section on Drug Design and Development, National Institute on Aging (NIA), National Institutes of Health (NIH), Bethesda, MD 20892-3711, USA; 3 Lieber Institute for Brain Development, Johns Hopkins Medical Campus, 855 N. Wolfe Street, Suite 300, Baltimore, MD 21205 USA; 4 Johnson & Johnson Pharmaceutical Research and Development, 1125 Trenton-Harbourton Road, Titusville, NJ, 08560 USA

**Keywords:** Apathy, Motivation, Chronic stress, Cholinergic, FosB, c-fos, Nucleus accumbens, Basal forebrain

## Abstract

**Background:**

Apathy is frequently observed in numerous neurological disorders, including Alzheimer's and Parkinson's, as well as neuropsychiatric disorders including schizophrenia. Apathy is defined as a lack of motivation characterized by diminished goal-oriented behavior and self-initiated activity. This study evaluated a chronic restraint stress (CRS) protocol in modeling apathetic behavior, and determined whether administration of an anticholinesterase had utility in attenuating CRS-induced phenotypes.

**Methods:**

We assessed behavior as well as regional neuronal activity patterns using FosB immunohistochemistry after exposure to CRS for 6 h/d for a minimum of 21 d. Based on our FosB findings and recent clinical trials, we administered an anticholinesterase to evaluate attenuation of CRS-induced phenotypes.

**Results:**

CRS resulted in behaviors that reflect motivational loss and diminished emotional responsiveness. CRS-exposed mice showed differences in FosB accumulation, including changes in the cholinergic basal forebrain system. Facilitating cholinergic signaling ameliorated CRS-induced deficits in initiation and motivational drive and rescued immediate early gene activation in the medial septum and nucleus accumbens.

**Conclusions:**

Some CRS protocols may be useful for studying deficits in motivation and apathetic behavior. Amelioration of CRS-induced behaviors with an anticholinesterase supports a role for the cholinergic system in remediation of deficits in motivational drive.

## Background

Apathy is characterized by severe loss of motivation to participate in activities, social withdrawal and emotional indifference [1]. Apathy shares some overlapping features with depression, but can be distinguished by lack of dysphoric symptoms including sadness, hopelessness and guilt [2,3]. Apathy is a frequent neuropsychiatric syndrome affecting up to 92% of individuals diagnosed with Alzheimer's disease (AD) [4-6], and up to 70% of those with Parkinson's disease (PD) [2,7-10]. Despite its prevalence, relatively little is known about the underlying neuropathology [10]. Stress exposure is an established risk factor for development of neuropsychiatric symptoms [11-14], and it has been established that animal models of chronic stress cause behavioral changes similar to symptoms of depression in humans [15]. Exposure to extreme forms of chronic stress, including time spent in prisoner of war and concentration camps as well as survival of the atomic bombing, has been documented to result in an apathetic syndrome[16-19]. For example, prisoners of the Korean War have been described as having a reactive syndrome that included extreme withdrawal of involvement and a paucity of emotion, which could not be explained by depression or psychosis, but was best characterized as "apathy" [16]. Visual observation of routine animal behavior led us to hypothesize that a 6 hr/d/> 21 d chronic restraint stress (CRS) protocol could be useful for modeling features of apathy. The objectives of the study were to 1) characterize the loss of motivation and initiative in CRS-exposed animals, 2) map long-term changes in neuronal activity in CRS-exposed animals and 3) determine whether facilitating the cholinergic system could ameliorate CRS-induced phenotypes.

## Methods

### Animals

**Figure 1:**
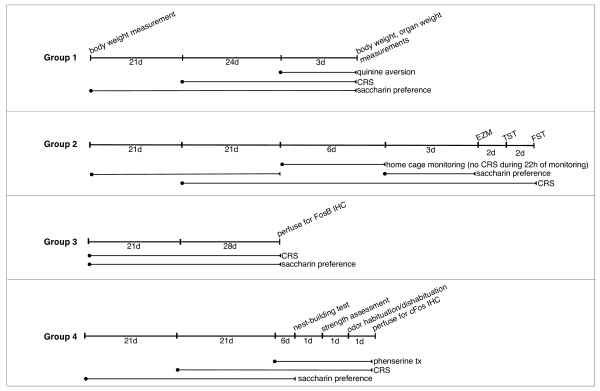
**Experimental design**. Group 1 (n = 10 control and n = 14 CRS-exposed for saccharin preference and quinine aversion; body and organ weight analysis performed in n = 8 for control versus CRS). Group 2 (for HCS, n = 8 control and n = 9 CRS-exposed; n = 8 control and n = 8 CRS-exposed for TST, FST and EZM). Group 3 (n = 6 Control and CRS-exposed). Group 4 (n = 12 Control and n = 16 CRS-exposed; CRS group further divided, n = 8 saline and n = 8 phenserine). For cFos immunohistochemistry (IHC) experiment control group further divided into n = 6 water exposure and n = 6 urine exposure; n = 6 saline/CRS/urine exposure and n = 6 phenserine/CRS/urine exposure were analyzed.

We used adult (8 wk) male C57BL/6J mice (Jackson Laboratories, Bar Harbor, ME, USA) that were double-housed in a standard mouse cage containing a metal divider splitting the cage into two separate compartments; each mouse retains an individual feeding compartment and water bottle. Mice were maintained under a 12:12 hour light-dark cycle (6:00 AM to 6:00 PM). All procedures were performed in accordance with guidelines set forth by the National Institute of Mental Health Animal Care and Use Committee in the Guide for the Care and Use of Laboratory Animals. Figure 1 gives an overview of the experimental design and groups used in the studies.

### Saccharin and quinine preference

21 d prior to CRS each mouse was given simultaneous access to two, dual-ball sipper-top bottles (Ancare, Bellmore, NY, USA): one with purified Milli-Q water and one containing 50 mg/L saccharine (Sigma Aldrich, St. Louis, MO, USA). Bottles were weighed and refilled every 3 d; positions were reversed at each change to prevent side bias. After 21 d, mice were divided into balanced groups with mice sharing a divider cage placed in the same experimental group. Individual animals with saccharin preference < 65% were excluded from the study (~3% of total animals used). For the quinine experiment, the saccharine solution was replaced with 15 mg/L quinine hydrochloride (Sigma Aldrich, St. Louis, MO, USA) solution for 3 d.

### Restraint stress

Mice were placed in 50 mL plastic conical tubes with holes cut at the tips to allow for unrestricted breathing and gauze was inserted in the remaining space. Mice were restrained for 6 h/d (10:00 AM-4:00 PM). All behavioral tests were performed before placement in restrainers (6 AM-10 AM).

### HomeCage scan

24 h after CRS, mice were placed into clean cages containing 100 mL sawdust bedding with food and water. Animals were monitored for 22 h using Sony digital cameras and CaptureStar video capturing software with infrared illumination during the dark phase. Automated video analysis of home cage behaviors was performed using HomeCageScan software (Clever Systems, Reston, VA). Behaviors were detected by utilizing information about the entire body of the animal, identifying animal body parts such as head, tail, forelimbs, hind limbs, upper/lower back, abdomen, etc., and using sequence data to automatically recognize and analyze animal behaviors in durations > 6 frames (30 frames/s).

### Odor Habituation/Dishabituation

We used a modified version of an odor discrimination task [20,21] to assess effects of CRS on response to an appetitive social stimulus, i.e female estrous urine. Cotton-tipped applicators were soaked with water and fastened to the roof of each cage such that mice must rear up to sniff. Duration of sniffing was measured over a 3 min presentation. This was repeated 3X, then replaced by 1% imitation vanilla, and finally by urine from an estrous-stage female. Contact with the applicator with an open mouth was considered chewing, and not scored as sniffing.

### Nest building

Old nesting material was removed, and two unused nestlets (paper-based nesting material compacted into white squares) [2 g/each] were placed on the cage floor. After 4 h, nestlet material that had not been either shredded or incorporated into a nest was weighed.

### Tail suspension test

Mice were suspended by their tails for 6 min using a 15 cm piece of lab tape wrapped around the tip of the tail. Sessions were videotaped and later scored by an observer blinded to groups. Any significant movement of the body or the limbs was considered as mobility.

### Forced swim test

Transparent plexiglass cylinders, 25 cm tall × 12 cm diameter were filled with 30°C water to ~21 cm so mice were not able to touch the floor or escape. Mice were placed in the water for 6 min, videotaped and later analyzed with Clever Systems Forced Swim Test Scan (Clever Sys Inc., Leesburg, VA, USA). At the end of each session, mice were dried with a paper towel and returned to their home cage. Water was replaced for each trial.

### Elevated zero maze

The ring-shaped platform consisted of two walled (white Plexiglas) sections separated by open sections of equal length. Each mouse was placed such that it was in an open section, directly facing a walled section. Activity was video-tracked for 5 min and analyzed using Clever Systems TopScan (Clever Sys Inc., Leesburg, VA, USA).

### Drug treatments

Phenserine ((-)-N-phenylcarbamoyleseroline) was synthesized as a water-soluble (L)-tartrate salt (> 99.9% optical and chemical purity)[22]. CRS-exposed mice were administered either 0.9% saline or phenserine (1 mg/kg, i.p) in the evening after the CRS session and again in the morning, 1 h prior to behavioral experiments. Animals within the CRS group were randomly divided into the continued CRS group and the CRS/phenserine group.

### Tissue preparation

Animals were anaesthetized under isofluorane and transcardially perfused with 50 mL of 4% paraformaldehyde (PFA) in phosphate buffered saline (PBS). Brains were removed from the skull and postfixed overnight at 4°C in 4% PFA/PBS, then transferred to 30% sucrose/PBS for 72 h for cryopreservation. Brains were mounted on a freezing stage (Physitemp Instruments, Inc., Clifton, NJ) set to -25°C and coronal sections (50 μm) were cut using a sliding microtome (Leica, Germany) and collected in PBS containing 0.015 M sodium azide.

### Immunohistochemistry

Every 6th (prefrontal cortex and brainstem) or 12th (hypothalamus, hippocampus) section was rinsed free-floating in PBS/0.5% Tween-20. Non-specific binding was blocked with 3% normal goat serum for 30 min. Sections were incubated with an anti-fos B antibody directed against the N-terminus, which detects both the full-length FosB as well as its truncated form, deltaFosB (sc-48, rabbit IgG, 1:500, Santa Cruz Biotechnology, Santa Cruz, CA) or with an anti-c-fos antibody (PC38, 1:1000, Calbiochem) for 24 h at 4°C. Sections were rinsed in PBS/0.5% Tween-20, incubated for 2 h at room temperature with biotinylated goat anti-rabbit secondary antibody (Vector Laboratories, Burlingame, CA). Endogenous peroxidase activity was blocked using 0.3% hydrogen peroxide for 30 min. The HRP-DAB reaction was carried out using an avidin/biotin peroxidase complex (VectaStain ABC Kit, Vector Laboratories). Sections were incubated in ABC for 1 hr and DAB-cobalt (Sigma, St. Louis MO) for 3 min. They were then mounted on SuperFrost-Plus treated slides (Fisher Scientific, Pittsburgh, PA), air-dried, Nissl stained, dehydrated with alcohol rinses, cleared with CitriSolv, and coverslipped with Permount.

### Image analysis

Section images were captured using a Leica DMRB light microscope equipped with a CoolSNAP digital camera and IPLab software. Cell density in the hippocampal dentate gyrus, nucleus accumbens, and cortical areas were analyzed using ImageJ. Cells in thalamic, hypothalamic, and septal nuclei were manually counted by a blinded individual. Anatomical boundaries were determined by using the Franklin and Paxinos mouse brain atlas [23].

### Statistical analysis

Data are presented as group means ± standard error of the mean (SEM). As appropriate, Student's *t*-test, one-way ANOVA with Newman Keul's *post hoc *or two-way ANOVA with Bonferroni *post hoc *were performed using GraphPad Prism 5. Statistical significance was defined as p < 0.05.

## Results

### Motivational deficits following chronic stress

**Figure 2:**
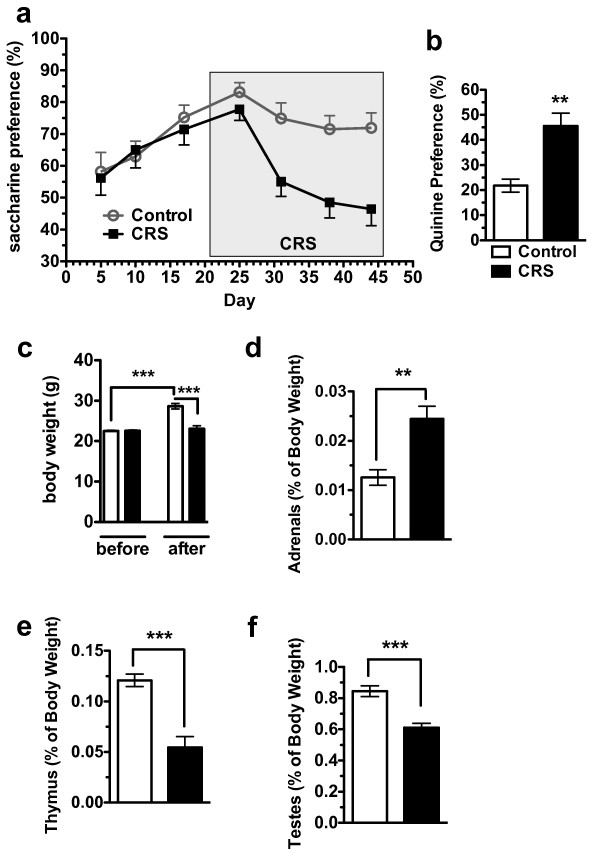
**CRS-exposed mice exhibit a decreased appetitive response, and fail to avoid a mildly aversive stimulus**. (**a**) Preference ratios for saccharin versus water in control and CRS-exposed mice in Group 1 (see Figure 1). Shaded area denotes CRS exposure. (**b**) Preference ratio for quinine versus water in control and CRS-exposed mice. (**c**) Control animals gain significantly more weight than CRS-exposed animals over the course of the experiment, while CRS animals do not gain a significant amount of weight and weigh significantly less than controls after CRS-exposure. (**d**) Increased adrenal weight in CRS-exposed animals; presented as % of total body weight. CRS exposure leads to decreased thymus weight. (f) CRS exposure leads to a decreased testes weight. Results here and in subsequent figures are reported as mean ± SEM; * = *p *< 0.05, ** = *p *< 0.01 and *** = *p *< 0.001.

Animals exposed to chronic stress consistently show deficits in the sucrose and/or saccharin preference test, findings which have contributed to establishing these tests as analogs of anhedonia [24-26]. A significant decrease in saccharin preference was found in CRS-exposed animals (n = 14) when compared to control animals (n = 10) beginning ~17 d after CRS initiation [ANOVA *F*_1,311 _= 17.33, *P *< 0.0001 for CRS treatment; *F*_6,311 _= 5.59, *P *< 0.0001 for time; *F*_6,311 _= 2.63, *P *= 0.0166 for time-CRS interaction; Bonferroni post-test, *P *> 0.05 at d5, d10, d17, d25 and d31, *P *< 0.05 at d38 and *P *< 0.01 at d44] (Figure 2a). Directly following the last measurement for saccharin preference we changed to a 2-bottle preference test between water and mildly bitter quinine solution (Figure 1, Group 1). As expected, control mice avoided the quinine, while CRS-exposed animals drank at levels close to chance [Student's *t*-test, *P *= 0.0012) (Figure 2b). To validate physiological markers of chronic stress we measured total body weight as well as weights of the adrenals, thymus and testes. Body weights of non-stressed animals (n = 8) significantly increased over the experiment while body weights of CRS-exposed animals (n = 8) did not [ANOVA *F*_1,28 _= 28.79, *P *< 0.0001 for CRS treatment; *F*_1,28 _= 30.79, *P *< 0.0001 for time; *F*_1,28 _= 30.54, *P *< 0.0001 for time-CRS interaction; Bonferroni post-test, *P *< 0.001 for Control versus CRS after CRS exposure] (Figure 2c). As expected, there was a significant increase in adrenal weight [*F*_7,6 _= 3.097, Student's *t*-test *P *= 0.0022] (Figure 1d) and significant decreases in both thymus [*F*_7,7 _= 2.186, Student's *t*-test *P *< 0.0001] (Figure 2e) and testes [*F*_7,7 _= 1.452, Student's *t*-test *P *= 0.0001] (Figure 2f) weight in CRS-exposed animals.

**Figure 3:**
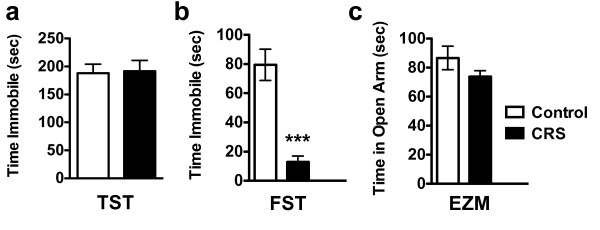
**CRS-exposure does not affect depressive- and anxiety- like behavior**. (**a**) Control and CRS-exposed animals spend a similar amount of time immobile in the tail suspension test. (**b**) CRS-exposed animals spend significantly less time immobile in the FST. (**c**) CRS-exposed animals do not differ in the amount of time spent in the open portion of an elevated zero maze.

The decrease in initiative to both approach an appetitive stimulus and avoid an aversive stimulus indicates a potential deficit in motivation. Chronic stress models have frequently been used to model symptoms of anhedonia, and these models consistently reveal related deficits in other depressive- and anxiety-like behaviors. We tested CRS-exposed animals (n = 8) as compared to control animals (n = 8) in two measures of behavioral despair, the tail suspension test (TST) and the forced swim test (FST). We observed no difference in immobility times in the TST [*F*_7,7 _= 1.385, Student's *t*-test *P *= 0.8802] (Figure 3a), but saw a significant decrease in immobility times in the FST [*F*_7,7 _= 5.994, Student's *t*-test *P *< 0.0001] (Figure 3b). This result is likely confounded by decreased body-weights in CRS-exposed animals (Figure 2c). We also tested CRS-exposed animals in the elevated zero maze (EZM) where an increase in time spent in the open portion of the maze indicates decreased anxiety-like behavior. Control and CRS-exposed animals spent similar amounts of times in the open portions of the maze [*F*_7,7 _= 3.854, Student's *t*-test *P *= 0.1749] (Figure 3c).

### Changes in home cage behavior following chronic stress

To better understand the full range of effects of CRS on normal home cage activity, we utilized automated behavioral recognition software, HomeCage Scan (CleverSys Inc., Reston, VA, USA), to monitor home cage activity for 22 h (Figure 1, Group2). We began recording 24 h following CRS, and analyzed the following behaviors: rear up, hang cuddled, drink, eat, groom, sleep, chew, twitch, sniff, remain low and walk slowly (software definitions in Table 1). Prior to experimentation, accuracy of the software in analyzing all behaviors was verified and calibrated to experienced hand-scorers.

**Table 1:** Software definitions for scoring HomeCage Scan behaviors

Behavior	Software Definition
**Rear Up**	Begins with the mouse lifting its front paws off the ground and standing on its hind legs. Rearing ends when the mouse comes back down and places one front paw back on the ground. Rearing may also include "partially reared" in which the mouse hunches its back and its front paws are off the ground, and is about halfway from being in the fully stretched, completely reared position.

**Hang Cuddled**	Both forelimbs and hindlegs are above the midpoint between the floor and the top of the cage.

**Drink**	Behavior starts when mouth is at level with the drinking spout. Behavior ends when mouth withdraws from the drinking spout. Sniffing behaviors directly before drinking are scored as drinking.

**Eat**	Snout is in the plane of the food compartment with minimal body and head movements. "Sniff" and "eat" are differentiated by the total time the snout remains in the food bin, with "sniff" being significantly less duration in the compartment. However, if the mouse sniffs directly before eating, the sniff behavior is scored as eating.

**Groom**	Mouse uses front paws to clean itself by rubbing over body and face in circular movements. Repetitive paw movements over a certain period of time are scored as groom.

**Sleep**	A minimum of 30 s of no significant movement of the mouse while it is in a non-rearing and non-hanging position is scored as sleep.

**Twitch**	Any movement occurring during sleep

**Sniff**	Body of mouse is stationary, but snout moves in a bobbing fashion. Scored as an exploratory behavior.

**Remain Low**	Prolonged inactivity of the mouse that is not scored by the software as any other behavior.

**Walk Slowly**	Mouse is moving across the cage and at least three legs are propelling it forward

**Figure 4:**
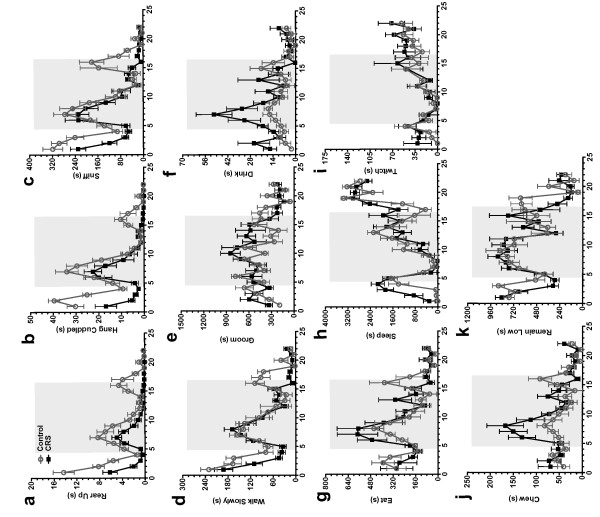
**Animals exposed to CRS show differences in the diurnal patterns of engaging in numerous home cage activities**. Data show 22 h behavior plots for (**a**) rearing up, (**b**) hanging-cuddled, (**c**) sniffing, (**d**) walking slowly, (**e**) grooming, (**f**) drinking, (**g**) eating, (**h**) sleeping, (**i**) twitching, (**j**) chewing, and (**k**) remaining low.

Our analysis revealed significant differences between control and CRS-exposed mice in total incidence as well as in diurnal patterns of numerous behaviors. CRS-exposed mice spent significantly less total time rearing, hanging cuddled, sniffing and walking slowly, but showed no difference in total time spent grooming drinking, eating, sleeping, twitching, chewing and remaining low (Table 2). In the first 41/2 hours prior to dark cycle onset, CRS-exposed mice spent less time rearing up (Figure 4a), hanging cuddled (Figure 4b), sniffing (Figure 4c), remaining low (Figure 4k) and walking slowly (Figure 4d). However, they spent more time drinking (Figure 4f) and resting (Figure 4h). It should be noted that differences in behavior between the control and CRS-exposed groups at experiment onset could reflect differences in novelty response since the cage was changed before beginning the recording. As expected there was a spike in most active behaviors (Figure 4a-d, f, g, j, k) at dark cycle initiation and a sharp drop in inactive behaviors (Figure 4h, i) in both groups. At this time, control and CRS-exposed animals were similar in sniffing (Figure 4c), walking slowly (Figure 4d) and remaining low (Figure 4k). However, the CRS-exposed group showed lower levels of rearing (Figure 4a) and hanging behavior (Figure 4b), but higher levels of drinking (Figure 4f), eating (Figure 4g) and chewing (Figure 4j) during the first ~4 hours of the dark phase. During the shift from dark to light (hours 15-18) CRS-exposed mice spent significantly more time resting than control mice (Figure 4h). As opposed to control mice, whose active behaviors showed a short, final peak right at the dark to light transition, CRS-exposed animals showed no peak in rearing (Figure 4a), hanging (Figure 4b), eating (Figure 4g), chewing (Figure 4j), walking slowly (Figure 4d), sniffing (Figure 4c) and remaining low (Figure 4k).

**Table 2:** Home-cage behavior analysis array of control versus CRS-exposed mice

	Control		CRS			
**Behavior**	**Mean (s)**	**SEM**	**Mean (s)**	**SEM**	***F value***	***P value***

Rear Up	81.08	9.21	28.99	4.26	3.12	< 0.0001

Hang Cuddled	268.7	18.13	118.7	25.23	2.9	0.01

Sniff	2783	230.2	1717	221.1	1.38	0.01

Walk Slowly	1918	143.4	1414	137.1	1.37	0.03

Groom	8820	771	10050	858.4	1.86	0.34

Drink	185.6	24.18	334.1	69.94	12.55	0.12

Eat	4451	503	4773	976.5	5.65	0.8

Sleep	27560	883	31670	1799	6.23	0.1

Twitch	667.2	75.62	776.3	58.71	1.11	0.27

Chew	930.4	139.9	1330	184.1	2.6	0.14

Remain Low	12120	810	11120	598.6	1.22	0.33

**Figure 5:**
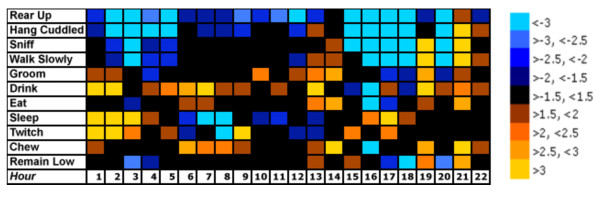
**Differences in specific home cage behaviors at discrete periods within the daily cycle**. Behavior-array analysis grid displaying fold increases and decreases between control and CRS-exposed mice. Each box represents an individual behavior and time point within the experiment. The intensity of color for each box represents the magnitude of behavioral change between control and CRS-exposed mice.

To examine overall trends in behavior, we created a behavior-array analysis grid displaying fold increases and decreases between control and CRS-exposed mice (Figure 5). Each box represents an individual behavior and time point within the experiment. The intensity of color for each box represents the magnitude of behavioral change between control and CRS-exposed mice. Overall, CRS-exposed mice displayed significant decreases in exploratory behaviors such as rear-up and sniff, as well as in locomotor behaviors including walk slowly, remain low, and hang cuddled for several hours directly prior to the onset of the dark cycle and before the onset of the light cycle (Figure 5), suggesting a decrease in initiation of non-essential activities. However, directly prior to dark cycle onset, inactive behaviors such as twitch and rest increased in the CRS mice.

### Neuronal activity mapping

**Figure 6:**
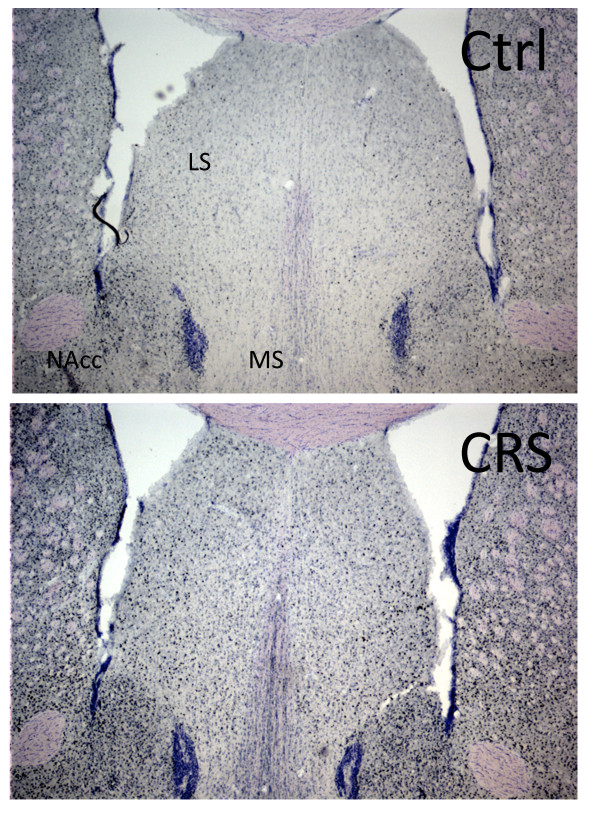
**Changes in deltaFosB immunoreactivity following CRS exposure**. Representative image showing increased deltaFosB immunoreactivity in the medial septum (MS) and lateral septum (LS) after exposure to CRS.

We used FosB immunohistochemistry to analyze neuronal activity patterns in control and CRS-exposed animals. Animals were killed 24 h following the last session of CRS and brains processed for FosB immunoreactivity (Figure 1, Group 3). The antibody used detects both full-length FosB as well as truncated deltaFosB, which gradually accumulates due to its high stability and long half-life. A recent report showed that in CRS-exposed rats (1 h/d/10 d), deltaFosB is the predominant Fos family protein induced and that the 35-37 kDa deltaFosB isoform is the only Fos family protein that remains elevated 24 h after the final stress exposure [27]. In agreement with a previous study [27], CRS-exposed mice (6 hr/d/28 d) showed significant increases in FosB immunoreactivity in the medial septum (MS)/nucleus of the vertical limb of the diagonal band (vDB) and the lateral septum (LS) (Table 3, Figure 6). Differing from this study [27], we observed significant increases in FosB immunoreactivity in hypothalamic regions including the arcuate nuclei and the paraventricular nucleus (PVN), but no differences in the prelimbic cortex (PrL), the infra-limbic cortex (IL) or the nucleus accumbens (NAcc). The differences between our results and those in previous studies may be explained by species and strain differences or from differences in CRS duration and protocol [27,28].

**Table 3:** deltaFosB immunoreactivity in control versus CRS-exposed mice

Brain Region	Control	CRS	Fold Difference		
	**Mean (Cells/pixel)**	**Mean (Cells/pixel)**	**CRS vs Control**	***F Value***	***P Value***

Dentate Gyrus	0	0	1.2891	1.533	0.11

Nucleus Accumbens	0	0	1.4205	2.061	0.07

Prelimbic Cortex	0	0	1.4308	8.433	0.05

Infralimbic Cortex	0	0	1.0169	1.328	0.93

Cingulate Cortex	0	0	1.0033	2.134	0.99

Visual Cortex	0	0	1.1538	1.295	0.25

	**Mean (cells counted)**	**Mean (cells counted)**			

Medial Septum/vDB	25.67	52.83	2.0580	1.794	0.01

Lateral Septum	568.7	1564	2.7501	2.510	< 0.0001

PVA	164.8	243	1.4745	2.718	0.22

PVN	34.17	125.3	3.6670	15.590	0

Arcuate	10	52.17	5.2170	12.710	0

SCN	5.83	2.5	0.4286	3.628	0.26

### Effects of cholinergic facilitation on motivation and apathetic behavior

DeltaFosB accumulation in the MS/vDB, one of the major basal forebrain cholinergic nuclei, suggests that this area undergoes significant changes in gene transcription and neuronal activity after CRS. This data, coupled with reports that treatment with acetylcholinesterase inhibitors (AChE-I) leads to remediation of apathetic behavior[10,29], led us to ask whether the CRS-induced behavioral deficits could be rescued by facilitating cholinergic signaling.

**Figure 7:**
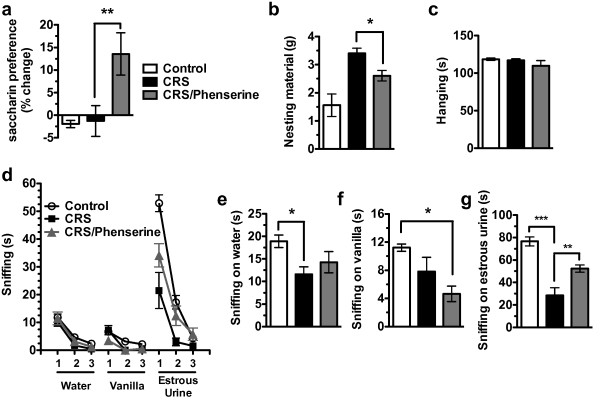
**Phenserine treatment results in partial recovery of CRS-induced deficits in motivation**. (**a**) Phenserine improves the CRS-induced saccharin preference deficit. (**b**) Phenserine improves the CRS-induced deficit in nesting behavior. (**c**) No change in muscle strength in CRS-exposed animals as determined by time the animal is able to hang from a wire rod. (**d**) Motivational drive to approach an appetitive stimulus is decreased by CRS, but partially rescued by phenserine administration. Habituation to the smell of a water-dipped Q-tip was measured over the course of three 3 min presentations by measuring time spent sniffing. Dishabituation and then habituation to a novel scent was assessed by measuring the time spent sniffing an imitation-vanilla dipped Q-tip for three consecutive 3 minute presentations and, lastly, an estrous urine dipped Q-tip. All groups showed normal habituation and dishabituation curves in response to the 3 stimuli. (**e**) Total time sniffing on the water smell is slightly decreased by CRS exposure. (**f**) Total time sniffing on the vanilla smell is slightly decreased in CRS-exposed animals administered phenserine. (**g**) Total time sniffing on estrous urine is robustly decreased by CRS exposure, but significantly improved by phenserine administration.

We administered phenserine, a centrally active and potent AChE-I, 21 d after initiating CRS. Phenserine was administered 2x/d/6 d in accord with its anticholinesterase half-life of 8.25 h before beginning behavioral analysis (Figure 1, Group 4) [30]. Preference ratios for Control animals were 74.5 ± 7.2 and preference ratios for CRS-exposed animals were 50.4 ± 4.7 prior to the phenserine study. Saline-injected CRS-exposed animals showed no improvement in saccharin preference, but CRS-exposed animals administered phenserine increased preference by ~13% over stress-induced depressed levels [ANOVA *F*_2,21 _= 6.505, *P *= 0.007; Newman-Keuls Multiple Comparisons for Control vs CRS *P *> 0.05, for Control vs CRS/Phenserine *P *< 0.05 and for CRS versus CRS/Phenserine *P *< 0.01] (Figure 7a). We next looked at the effect of phenserine on the motivation levels of CRS-exposed animals in a nest-building paradigm. Existing nests and nesting materials were removed, and animals were provided new nesting material. After 4 h, unused nesting material was measured. CRS-exposed animals showed significantly decreased motivation in nest building as determined by incorporation of less nestlet material, but phenserine treatment led to a significant improvement [ANOVA *F*_2,21 _= 11.00, *P *= 0.0001; Newman-Keuls Multiple Comparisons for Control vs CRS *P *< 0.001, for Control vs CRS/Phenserine *P *< 0.05 and for CRS versus CRS/Phenserine *P *< 0.05] (Figure 7b). We ruled out the possibility that the decreased nest-building could be a result of weakness in the CRS-exposed animals by performing a wire hang test to check for muscle strength [ANOVA *F*_2,21 _= 1.172, *P *= 0.3291] (Figure 7c).

To determine whether loss of motivational drive in the saccharin preference test transfers to an alternative appetitive stimulus, we measured the time spent sniffing on urine from a female estrous mouse in a modified odor habituation/dishabituation test. We first measured time spent sniffing a Q-tip dipped in water upon its initial presentation and then habituation to the smell on the 2nd and 3rd presentations. Next, we determined whether the animal showed normal dishabituation in response to presentation of a novel vanilla scent and then habituation to this scent on the 2nd and 3rd presentations. Lastly, we introduced female estrous urine and measured dishabituation and then habituation. Both control and CRS-exposed mice showed normal habituation and dishabituation curves in response to the 3 stimuli (Figure 7d). Animals exposed to CRS spent slightly less overall time sniffing on water [ANOVA *F*_2,21 _= 4.101, *P *= 0.0314; Newman-Keuls Multiple Comparisons for Control vs CRS *P *< 0.05, for Control vs CRS/Phenserine *P *> 0.05 and for CRS versus CRS/Phenserine *P *> 0.05] (Figure 7e), no difference in time spent sniffing on vanilla [ANOVA *F*_2,21 _= 5.46, *P *= 0.0149; Newman-Keuls Multiple Comparisons for Control vs CRS *P *> 0.05, for Control vs CRS/Phenserine *P *< 0.05 and for CRS versus CRS/Phenserine *P *> 0.05] (Figure 7f), and significantly less time sniffing on estrous urine [ANOVA *F*_2,21 _= 24.03, *P *< 0.0001; Newman-Keuls Multiple Comparisons for Control vs CRS *P *< 0.001, for Control vs CRS/Phenserine *P *< 0.01 and for CRS versus CRS/Phenserine *P *< 0.01] (Figure 7g).

**Figure 8:**
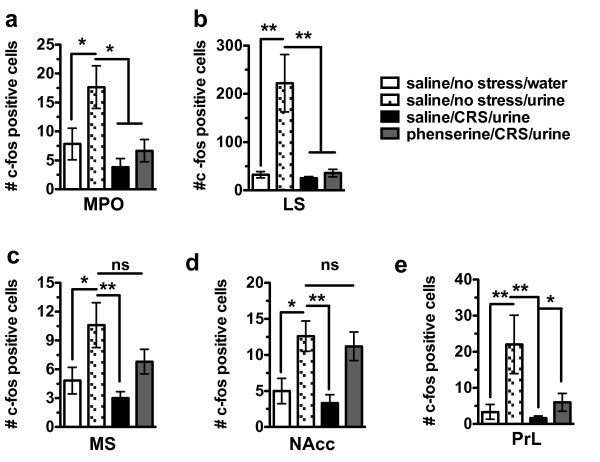
**Treatment with the AChE-I, phenserine provides a partial recovery for CRS-induced deficits in immediate early gene activation following exposure to a motivational stimulus**. (**a**) Control animals (no CRS), injected with saline and exposed to a Q-tip dipped in estrous urine (hatched bars) show significantly increased numbers of c-fos positive nuclei in the medial preoptic area (MPO) as compared to those exposed to a Q-tip dipped in water (white bars). CRS animals administered either saline (black bars) or phenserine (grey bars) do not show an increase in c-fos positive nuclei after sniffing on estrous urine. (**b**) Control animals (no CRS), injected with saline and exposed to a Q-tip dipped in estrous urine (hatched bars) show significantly increased numbers of c-fos positive nuclei in the LS as compared to those exposed to a Q-tip dipped in water (white bars). CRS-exposed animals administered either saline (black bars) or phenserine (grey bars) do not show an increase in c-fos positive nuclei after sniffing on estrous urine. (**c**) Control animals (no CRS), injected with saline and exposed to a Q-tip dipped in estrous urine (hatched bars) show significantly increased numbers of c-fos positive nuclei in the MS as compared to those exposed to a Q-tip dipped in water (white bars). CRS-exposed animals administered saline (black bars) do not show an increase in c-fos positive nuclei after sniffing on estrous urine. CRS-exposed animals administered phenserine (grey bars) partially recover the induction in c-fos positive nuclei following estrous urine exposure. (**d**) Control animals (no CRS), injected with saline and exposed to a Q-tip dipped in estrous urine (hatched bars) show significantly increased numbers of c-fos positive nuclei in the NAcc as compared to those exposed to a Q-tip dipped in water (white bars). CRS animals administered saline (black bars) do not show an increase in c-fos positive nuclei after sniffing on estrous urine. CRS-exposed animals administered phenserine (grey bars) fully recover the induction in c-fos positive nuclei following estrous urine exposure. (**e**) Control animals (no CRS), injected with saline and exposed to a Q-tip dipped in estrous urine (hatched bars) show significantly increased numbers of c-fos positive nuclei in the PrL as compared to those exposed to a Q-tip dipped in water (white bars). CRS animals administered either saline (black bars) or phenserine (grey bars) do not show an increase in c-fos positive nuclei after sniffing on estrous urine.

To better understand the brain regions phenserine may act on to mediate its behavioral effects in CRS-exposed animals, we used c-fos immunohistochemistry to examine neuronal activation patterns in response to presentation of estrous urine. For this experiment, animals were divided into 4 groups: saline-injected/non-CRS exposed animals that sniffed on a Q-tip dipped in water, saline-injected/non-CRS exposed animals that sniffed on a Q-tip dipped in estrous urine, saline-injected/CRS exposed animals that sniffed on a Q-tip dipped in estrous urine and phenserine-injected/CRS-exposed animals that sniffed on Q-tip dipped in estrous urine. Animals were allowed to sniff on the Q-tip for 3 min and killed 2 h later for c-fos immunohistochemistry. In non-stressed, saline-injected animals (n = 6), sniffing on estrous urine (hatched bars) as opposed to water (white bars) increases c-fos immunoreactivity in numerous brain regions including the medial preoptic area (MPO), LS, MS, NAcc and PrL (Figure 8a, b, c, d and 8e). CRS-exposed animals that were injected with saline (n = 6) (black bars) did not show the increase in c-fos immunoreactivity in the MPO, LS, MS, NAcc and PrL (Figure 8a, b, c, d and 8e) after sniffing on estrous urine. However, phenserine administration in CRS-exposed animals (n = 6) was capable of fully rescuing the response to estrous in the NAcc (Figure 8d) and partially rescuing the response in the MS (Figure 8c). ANOVA and post-hoc statistics for Figure 8 are provided in the accompanying Table 4.

**Table 4:** ANOVA table for statistics in Figure 8

Newman-Keul's post hoc								
**Brain Region**	***F value***	***P value***	**white vs hatched**	**white vs black**	**white vs gray**	**hatched vs black**	**hatched vs gray**	**black vs hatched**

MPO	5.03	*P < 0.05*	*P > 0.05*	*P > 0.05*	*P < 0.05*	*P < 0.05*	*P < 0.05*	*P < 0.05*

MPO	5.03	*P < 0.05*	*P > 0.05*	*P > 0.05*	*P < 0.05*	*P < 0.05*	*P < 0.05*	*P < 0.05*

LS	8.76	*P < 0.01*	*P > 0.05*	*P > 0.05*	*P < 0.01*	*P < 0.01*	*P < 0.001*	*P < 0.01*

MS	4.82	*P < 0.05*	*P > 0.05*	*P > 0.05*	*P < 0.01*	*P < 0.01*	*P > 0.05*	*P < 0.01*

NAcc	6.8	*P < 0.05*	*P > 0.05*	*P < 0.05*	*P < 0.01*	*P < 0.01*	*P > 0.05*	*P < 0.01*

## Discussion

A major objective of this study was to characterize CRS-induced deficits in motivational drive. As expected CRS-exposed animals lost their preference for saccharin, but also failed to avoid a bitter quinine solution (Figure 2a, b). A similar phenomenon has been reported in rhesus monkeys following maternal deprivation [31]. This study proposed that in addition to producing anhedonia, some chronic stress paradigms may decrease motivation for appetitive stimuli in general [31]. Deficits in the sucrose and saccharin preference tests have been reliably used as measures of anhedonia [24-26], which is defined as the inability to experience pleasure in previously enjoyable activities such as eating, exercising, socializing and sex [32-34]. The saccharin preference deficit coupled with the lack of quinine aversion may also indicate apathy, a lack of interest in surroundings, social withdrawal and loss of motivation and initiative [35,36]. Apathy, on its own, or when co-morbid with depression poses a challenge to clinicians due to their overlapping symptomatology and frequent co-occurrence [3]. Identifying apathy requires differentiation between loss of initiation versus loss of ability and emotional indifference versus a primary mood disturbance [7]. Modeling apathy is important since it does not respond similarly to treatment options for anhedonia [37,38]. Accordingly, despite their overlapping symptomatology, there is accumulating evidence that apathy and anhedonia may have different underlying alterations in brain circuits [3,39].

We show that loss of motivational drive in CRS-exposed animals in the saccharin preference test can transfer to a decrease in motivation for an alternative appetitive stimulus. First, we showed that CRS-exposed animals showed normal habituation and dishabituation to three different odors, confirming intact olfactory senses (Figure 7d). However, we observed a significant decrease in interest for estrous urine (Figure 7g), suggesting that these animals exhibit deficits in motivational drive. CRS-exposed animals also show lack of motivation in a nest-building paradigm [35,36] and decreases in home-cage exploratory behaviors. For example, we saw significant differences in total time spent rearing up, hanging cuddled and sniffing (Table 2), and in patterns of diurnal activity. Since alterations in sleep and circadian rhythms play a critical role in the pathophysiology of numerous neuropsychiatric disorders[33,40,41], the ability to model circadian alterations is a useful experimental tool.

It has been suggested that apathy may reflect an interaction between cholinergic deficiency and subsequent neurological changes in limbic regions [42]. Thus, we asked whether deltaFosB accumulation in the MS/vDB, which constitutes the major cholinergic projection to the hippocampal formation, cingulate cortex and the hypothalamus [29] could be influencing cholinergic signaling. AChE-I treatment reduces incidence of apathy and improves functioning in patients who present with cholinergic disturbances in limbic and paralimbic cortices [10,29], and restoration of function in these brain regions may underlie the behavioral response to AChE-Is [9,43]. In AD, functional loss is thought to be a consequence of neuronal loss in cholinergic nuclei, and it has previously been reported that CRS can result in hippocampal atrophy [44]. However, it appears that cholinergic function in our model may be altered via changes resulting from alterations in plasticity as opposed to neuronal loss because levels of the cholinergic cell marker p75^NTR ^are unchanged between control and CRS-exposed animals and there are no appreciable changes in regional volume between control and CRS-exposed animals (KM and RJS, unpublished observations).

It is also possible that the behavioral effects of phenserine in our model result from activation of cholinergic interneurons in areas implicated in motivation and reward. For example, it has been shown that the AChE-Is galantamine and donepezil lead to increased dopamine release in NAcc [45,46]. Control animals show a robust increase in immediate early gene activation in the NAcc after being exposed to a motivating stimulus, i.e. estrous urine (Figure 8d), but this increase is lost in CRS-exposed animals. However, phenserine administration rescued this deficit, suggesting that cholinergic facilitation may restore dopaminergic function in the CRS-exposed NAcc. This restorative function could contribute to phenserine's role in behavioral rescue of motivational drive in CRS-exposed animals (Figure 7a, b, d).

The focus of the experiments with phenserine was to determine whether an anticholinesterase had utility in reversing selected CRS-induced phenotypes rather than determining the effect of the drug in a naïve population. However, it remains a caveat of our studies that our study did not include a control group to look at the effects of phenserine in a non-CRS exposed population. Thus, it is possible that the effects of phenserine may not be limited to animals exposed to CRS, but may also have similar effects on a control population.

## Conclusions

This CRS protocol resulted in behaviors reflecting motivational loss and diminished emotional responsiveness as well as changes in deltaFos B accumulation in brain regions that could affect normal cholinergic signaling. Facilitating the cholinergic system results in partial rescue of CRS-induced impairments to initiation and motivational drive. Our study provides support for further study of utilizing AChE-Is and cholinergic mimetics in neuropsychiatric symptoms of motivational loss and apathetic behavior.

## Abbreviations

CRS: Chronic restraint stress; AD: Alzheimer's disorder; TST: Tail suspension test; FST: Forced swim test; EZM: Elevated zero maze; MS: Medial septum; vDB: Vertical limb of the diagonal band; LS: Lateral septum; PVN: Paraventricular nucleus; NAcc: Nucleus accumbens; ANOVA: Analysis of variance; MPO: Medial preoptic area; PrL: Prelimbic area

## Competing interests

Keri Martinowich, Kathleen M. Cardinale, Michael Hsu, Nigel H. Greig and Robert J. Schloesser report no biomedical financial interests or potential conflicts of interest. Husseini K. Manji is a paid employee of Johnson and Johnson Pharmaceutical Research.

## Authors' contributions

KM designed the studies, performed research, analyzed data and wrote the manuscript. KMC performed research and analyzed data. RJS designed the studies, performed research and analyzed data. MH performed research and analyzed data. NHG designed the studies and synthesized phenserine. HKM designed the studies. All authors read and approved the final manuscript.
